# Retrospective cohort study based on the MIMIC-IV database: analysis of factors influencing all-cause mortality at 30 days, 90 days, 1 year, and 3 years in patients with different types of stroke

**DOI:** 10.3389/fneur.2024.1516079

**Published:** 2025-01-07

**Authors:** Xuehui Fan, Jing Xu, Ruixue Ye, Qiu Zhang, Yulong Wang

**Affiliations:** Department of Rehabilitation Medicine, The First Affiliated Hospital of Shenzhen University/The Second People’s Hospital of Shenzhen, Shenzhen, China

**Keywords:** stroke, prognostic factors, clinical characteristics, machine learning, mortality risk prediction

## Abstract

**Objective:**

This study aims to evaluate key factors influencing the short-term and long-term prognosis of stroke patients, with a particular focus on variables such as body weight, hemoglobin, electrolytes, kidney function, organ function scores, and comorbidities. Stroke poses a significant global health burden, and understanding its prognostic factors is crucial for clinical management.

**Methods:**

This is a retrospective cohort study based on data from the MIMIC-IV database, including stroke patients from 2010 to 2020. A total of 5,110 patients aged 18 and older were included in the study. The exposure variables included body weight and hemoglobin levels, while the outcome variables were the 30-day, 90-day, 1-year, and 3-year mortality risks. Covariates included electrolyte levels, kidney function, organ function scores, and comorbidities. Random forest and gradient boosting tree models were employed for data analysis to assess mortality risk.

**Results:**

Kaplan–Meier survival analysis showed that ischemic stroke patients had the highest 30-day mortality rate at 8.5%, with only 20% 1-year survival. Traumatic subarachnoid hemorrhage patients had the best prognosis, with a 1-year survival rate of 60%. Multivariable Cox regression analysis revealed that each 1-point increase in the Charlson Comorbidity Index raised the 1-year and 3-year mortality risks by 1.39 times (95% CI: 1.10–1.56) and 1.44 times, respectively. Each 1-point increase in the SOFA score increased the 30-day, 90-day, 1-year, and 3-year mortality risks by 2.11 times, 2.03 times, and 1.84 times, respectively. Additionally, lower hemoglobin levels were significantly associated with increased mortality, with 30-day, 90-day, and 1-year mortality risks increasing by 3.33 times, 3.34 times, and 4.16 times, respectively (*p* < 0.005). Age ≥ 71 years, longer hospital stays, and organ dysfunction were also significant factors affecting mortality.

**Conclusion:**

This study highlights the critical role of stroke type, comorbidity index, SOFA score, hemoglobin levels, and length of hospital stay in stroke prognosis. These findings provide valuable insights for clinical risk assessment and the development of individualized treatment strategies, which may improve the management and outcomes of stroke patients. The predictive model constructed effectively assesses mortality risks in stroke patients, offering support for future clinical practice.

## 1 Introduction

Stroke is a severe disease that poses a significant threat to human health. Globally, approximately 17 million people experience a stroke each year, with around 6 million deaths (1). In China, the incidence and mortality rates of stroke are also alarmingly high, with about 2.6 million new cases annually and over 1 million deaths (2). Different types of stroke, such as ischemic stroke, hemorrhagic stroke, and subarachnoid hemorrhage, exhibit variations in incidence and prognosis. For example, in China, ischemic stroke accounts for 69.6% of all strokes, hemorrhagic stroke for 30.0%, and subarachnoid hemorrhage for 0.4%, with 30-day case fatality rates of 12.5, 37.5, and 45.9%, respectively (3).

Several factors, including age, Charlson Comorbidity Index (CCI), Sequential Organ Failure Assessment (SOFA) score, length of hospital stay, Glasgow Coma Scale (GCS) score at admission, gender, race, laboratory results, and vital signs, may influence stroke prognosis. Age is a key risk factor, with the incidence and mortality rates of stroke rising significantly with increasing age (4). The Charlson Comorbidity Index reflects the severity of underlying diseases and has been found to negatively correlate with stroke prognosis (5). The SOFA score, which assesses the extent of organ dysfunction, is also significantly associated with stroke outcomes (6). Additionally, lower GCS scores at admission, prolonged hospital stays, and certain abnormal laboratory indicators suggest a poor prognosis (7–9).

Although substantial research has explored the relationship between these factors and stroke prognosis, there are limitations in existing evidence. First, most studies focus on a single type of stroke, with few covering different subtypes. Second, many studies are single-center with small sample sizes, limiting their generalizability to national or global levels. Furthermore, most previous studies have focused on short-term prognosis (30 or 90 days), with limited cohort studies following patients for longer periods. Therefore, a large-scale, multicenter retrospective cohort study is needed to comprehensively assess the impact of these factors on the long-term prognosis of patients with different types of stroke.

This study aims to conduct a retrospective cohort analysis using the MIMIC-IV database, covering stroke patients (including ischemic stroke, hemorrhagic stroke, and subarachnoid hemorrhage) treated between January 1, 2010, and December 31, 2020. We will evaluate the association between factors such as age, Charlson Comorbidity Index, SOFA score, length of hospital stay, GCS score at admission, gender, race, laboratory results, and vital signs, with 30-day, 90-day, 1-year, and 3-year all-cause mortality risk, providing more comprehensive evidence for clinical practice.

## 2 Methods

### 2.1 Study design

This is a retrospective cohort study utilizing data from the MIMIC-IV (Medical Information Mart for Intensive Care IV) database. The study aims to evaluate the all-cause mortality rates at 30 days, 90 days, 1 year, and 3 years among patients with different types of stroke. The stroke types analyzed include cerebral infarction, intracerebral hemorrhage, non-traumatic subarachnoid hemorrhage, and traumatic subarachnoid hemorrhage (10, 11).

### 2.2 Study population

A total of 5,110 patients were included in this study, with 71.8% diagnosed with cerebral infarction, 14.3% with intracerebral hemorrhage, 5.6% with non-traumatic subarachnoid hemorrhage, and 8.3% with traumatic subarachnoid hemorrhage.

#### 2.2.1 Inclusion criteria

Adult patients (age ≥ 18 years) with a confirmed diagnosis of one of the four stroke types (cerebral infarction, intracerebral hemorrhage, non-traumatic subarachnoid hemorrhage, or traumatic subarachnoid hemorrhage).

Patients with complete follow-up information for mortality outcomes at 30 days, 90 days, 1 year, and 3 years.

#### 2.2.2 Exclusion criteria

Patients with missing data on key demographic or clinical variables (e.g., age, gender, Charlson comorbidity index, or mortality status).

Patients with multiple strokes during their hospital stay were excluded if the stroke type could not be clearly identified, or if follow-up data were incomplete.

### 2.3 Variables

The primary outcome of interest was all-cause mortality at 30 days, 90 days, 1 year, and 3 years following stroke onset. Covariates included demographic variables (age, gender, race), clinical indicators (Charlson comorbidity index, Sequential Organ Failure Assessment [SOFA] score, Glasgow Coma Scale [GCS] at admission), and hospital-related variables (length of hospital stay, use of vasopressors such as epinephrine, dobutamine, and dopamine).

### 2.4 Data source

The data used in this study were extracted from the publicly available MIMIC-IV database, which contains de-identified health-related information for over 40,000 critical care patients admitted to the Beth Israel Deaconess Medical Center between 2008 and 2019. MIMIC-IV includes detailed clinical data, such as vital signs, laboratory results, and hospital outcomes, and has been extensively validated for research purposes.

### 2.5 Data processing

Data Import and Cleaning: Data were imported from the CSV file merged_output_with_3_year_death_info.csv. Filtering was performed to retain only patients diagnosed with cerebral infarction, intracerebral hemorrhage, non-traumatic subarachnoid hemorrhage, or traumatic subarachnoid hemorrhage. Missing values in key variables were handled using listwise deletion.

#### 2.5.1 Variable categorization

Continuous variables (e.g., age, Charlson comorbidity index, SOFA score, hospital stay duration) were standardized where appropriate.

Categorical variables included stroke type, GCS level, gender, race (categorized), and use of vasopressors. Stroke types served as the primary grouping variable for the analyses.

### 2.6 Statistical analysis

#### 2.6.1 Descriptive statistics

Descriptive statistics were used to summarize baseline characteristics. Continuous variables were tested for normality using the Shapiro–Wilk test. For normally distributed variables, mean ± standard deviation (SD) was reported, whereas for non-normally distributed variables, median and interquartile range (IQR) were used. Differences between stroke types were assessed using ANOVA for normally distributed variables and the Kruskal-Wallis test for non-normally distributed variables. Categorical variables were presented as frequencies and percentages, with chi-square tests employed to evaluate group differences.

#### 2.6.2 Mortality rate calculation

Mortality rates at 30 days, 90 days, 1 year, and 3 years were calculated for each stroke type by computing the proportion of patients who died at each time point, divided by the total number of patients in each stroke group. The rates were expressed as percentages, and group comparisons were performed using chi-square tests.

#### 2.6.3 Multivariable logistic regression

To explore the association between predictor variables and mortality at each time point (30 days, 90 days, 1 year, 3 years), multivariable logistic regression models were fitted. Covariates included demographic information, clinical scores (Charlson comorbidity index, SOFA score), and hospital-related factors (length of hospital stay, use of vasopressors). Standardized coefficients and odds ratios (OR) with 95% confidence intervals (CI) were reported. Model fit was assessed using the Hosmer-Lemeshow goodness-of-fit test.

#### 2.6.4 Survival analysis

Kaplan–Meier survival curves were generated to estimate the survival probabilities over time for each stroke type. The survival time was defined as the number of days from stroke diagnosis to death or the end of follow-up, and the event of interest was all-cause mortality. The log-rank test was used to compare survival curves across stroke types. Additionally, Cox proportional hazards models were fitted to adjust for potential confounders, including age, gender, Charlson comorbidity index, and SOFA score. Hazard ratios (HR) with 95% confidence intervals were reported.

#### 2.6.5 Training and validation of machine learning models

In this study, random forest and gradient boosting tree models were employed for data analysis. The training and validation process consisted of the following steps:

##### 2.6.5.1 Dataset splitting

The dataset was randomly divided into a training set (70%) and a testing set (30%) to ensure robust model evaluation.

##### 2.6.5.2 Hyperparameter optimization

Optimal hyperparameters were determined using Grid Search combined with 5-fold cross-validation to enhance model performance.

##### 2.6.5.3 Performance evaluation

Model performance was assessed using standard metrics, including the area under the receiver operating character ristic curve (AUC), accuracy, sensitivity, and specificity.

##### 2.6.5.4 Feature importance analysis

The contribution of individual variables to the model’s predictions was quantified using SHAP (Shapley Additive Explanations) values, providing insights into the relative importance of each feature.

### 2.7 Stratified survival analysis

To further explore the impact of key clinical variables on survival, Kaplan–Meier analyses were stratified by age group, Charlson comorbidity index, and SOFA score. Log-rank tests were performed within each stratum to assess the statistical significance of differences in survival.

### 2.8 Ethical considerations

The MIMIC-IV database is a de-identified, publicly available dataset, and its use for research has been approved by the Institutional Review Board (IRB) of the Massachusetts Institute of Technology (MIT). As the data are anonymized, the requirement for informed consent was waived.

## 3 Results

### 3.1 Analysis of clinical characteristics of stroke patients

#### 3.1.1 Comparison of clinical characteristics

This study compared the clinical characteristics of four different types of stroke patients (Table 1). The results showed that the average age of patients with cerebral infarction was 72.28 years (IQR: 18.92), which was higher than other stroke types. The average age of patients with intracerebral hemorrhage was 70.04 years, while non-traumatic subarachnoid hemorrhage patients had the lowest average age of 60.59 years. Additionally, both the Charlson Comorbidity Index for cerebral infarction and intracerebral hemorrhage patients was 6.00 (IQR: 3.00), whereas for non-traumatic and traumatic subarachnoid hemorrhage patients, it was 4.00 (IQR: 3.00 and 4.00), indicating a heavier comorbidity burden in the former two groups. In terms of SOFA scores, both the cerebral infarction group and the traumatic subarachnoid hemorrhage group had an average score of 4.00 (IQR: 4.00), reflecting a higher risk of organ dysfunction, while the non-traumatic subarachnoid hemorrhage group had a score of only 2.00 (IQR: 4.00), indicating less severe organ dysfunction. The length of hospital stay results further indicated that non-traumatic subarachnoid hemorrhage patients had the longest hospital stays (4.49 days, IQR: 9.92), while patients with cerebral infarction had the shortest stay, averaging only 1.88 days (IQR: 2.97). These results emphasize significant differences in age, comorbidity burden, organ dysfunction, and hospitalization management time among different types of stroke patients, providing important evidence for clinical management and treatment strategies.

**Table 1:** Comparison of clinical characteristics by stroke type.

Variable	Cerebral infarction	Intracerebral hemorrhage	Non-traumatic subarachnoid hemorrhage	Traumatic subarachnoid hemorrhage
Age	72.28 [IQR: 18.92]	70.04 [IQR: 20.97]	60.59 [IQR: 21.42]	66.85 [IQR: 27.79]
Charlson comorbidity index	6.00 [IQR: 3.00]	6.00 [IQR: 3.00]	4.00 [IQR: 3.00]	4.00 [IQR: 4.00]
SOFA	4.00 [IQR: 4.00]	3.00 [IQR: 4.00]	2.00 [IQR: 4.00]	4.00 [IQR: 4.00]
Hospital stay duration (days)	1.88 [IQR: 2.97]	2.91 [IQR: 4.76]	4.49 [IQR: 9.92]	2.23 [IQR: 4.34]

#### 3.1.2 Consciousness status and gender distribution

This study conducted an in-depth analysis of the clinical characteristics of different stroke types (Table 2). The results showed that patients with cerebral infarction had higher Glasgow Coma Scale (GCS) scores upon admission compared to other types of patients. Specifically, in the cerebral infarction group, 2,722 patients were normal, 630 had mild coma, 191 had moderate coma, and 126 had severe coma, while the numbers of normal and mild coma patients in the intracerebral hemorrhage group were 494 and 176, respectively, suggesting that patients with cerebral infarction generally maintained better consciousness. Furthermore, gender analysis revealed that the proportion of males (1,939) in the cerebral infarction group was higher than that of females (1,730).

**Table 2:** Clinical characteristics by diagnosis category.

Variable	Cerebral infarction	Intracerebral hemorrhage	Non-traumatic subarachnoid hemorrhage	Traumatic subarachnoid hemorrhage
Admission_GCS_Level (normal, mild coma, moderate coma, severe coma)	2,722, 630, 191, 126	494, 176, 37, 23	219, 50, 8, 8	305, 93, 17, 11
Gender (female, male)	1730, 1939	334, 396	168, 117	153, 273
Hospital_Expire_Flag (survive, death)	3,355, 314	590, 140	231, 54	382, 44
Epinephrine (not used, used)	3,475, 194	720, 10	276, 9	418, 8
Dobutamine (not used, used)	3,605, 64	728, 2	283, 2	426, 0
Dopamine (not used, used)	3,608, 61	729, 1	285, 0	418, 8
Categorized_Race (non-white, white)	1,364, 2,305	289, 441	123, 162	181, 245

GCS score: Glasgow Coma Scale, used to assess the level of consciousness in patients. The grading includes: Normal (15 points), Mild Coma (13–14 points), Moderate Coma (9–12 points), and Severe Coma (≤8 points).

Regarding hospitalization outcomes, the number of survivors among cerebral infarction patients was 3,355, demonstrating a favorable prognosis compared to 314 deaths. In contrast, the intracerebral hemorrhage group had 590 survivors and 140 deaths, reflecting notable differences in prognosis between different types of stroke. In terms of medication usage, the proportion of patients using adrenaline in the cerebral infarction group was lower than in other groups, with only 194 patients using it, while only 10 patients in the intracerebral hemorrhage group received adrenaline. Additionally, the usage rates of dopamine and dobutamine also showed differences. Finally, racial analysis indicated that the proportion of Caucasian patients in the cerebral infarction cohort (2,305) was higher than that of non-Caucasians (1,364). These results provide important empirical evidence for the clinical management and treatment strategies for stroke patients.

#### 3.1.3 Survival rates and treatment outcomes

**Figure 1:**
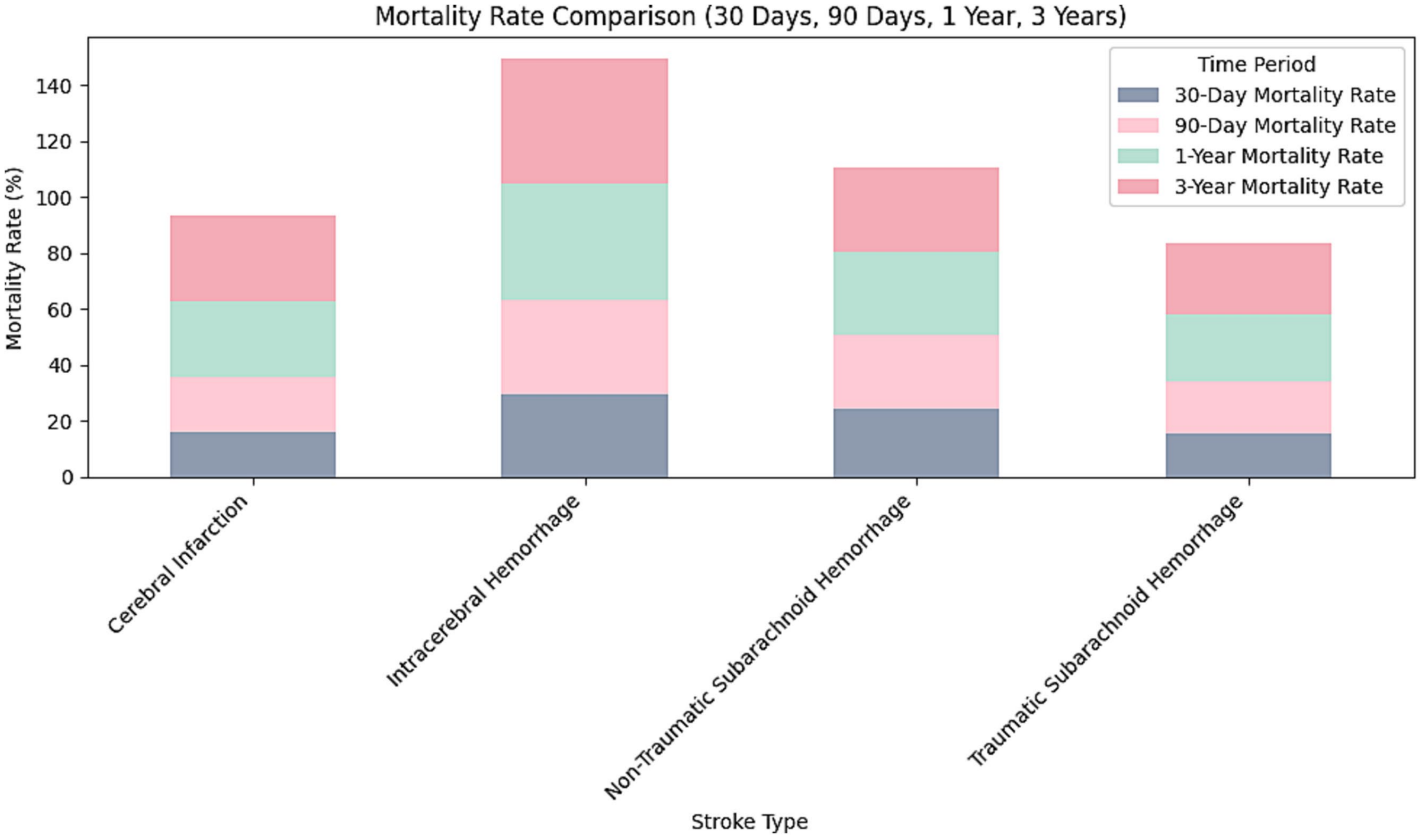
Mortality rate comparison for different stroke types (30 Days, 90 Days, 1 Year, 3 Years). This bar chart compares mortality rates at 30 days, 90 days, 1 year, and 3 years for four stroke types: cerebral infarction, intracerebral hemorrhage, non-traumatic subarachnoid hemorrhage, and traumatic subarachnoid hemorrhage. Each bar is divided into four segments, representing the mortality rate for each time period. Intracerebral hemorrhage shows the highest cumulative mortality, while cerebral infarction has the lowest.

This study analyzed the survival rates of different types of stroke patients (Table 3; Figure 1). The results showed that the survival rate of patients with cerebral infarction was the lowest, with only about 20% surviving at 350 days. This finding is particularly interesting and may be attributed to the irreversible damage caused by ischemia, the higher prevalence of comorbidities such as hypertension and diabetes, and the limitations of current treatments like thrombolysis and thrombectomy. In contrast, patients with traumatic subarachnoid hemorrhage had the highest survival rate, with about 60% surviving at the end of follow-up. The survival rates for patients with intracerebral hemorrhage and non-traumatic subarachnoid hemorrhage fell between the two, at approximately 30 and 50%, respectively, at 350 days. Log-rank test analysis indicated a significant difference between the cerebral infarction and intracerebral hemorrhage groups (*p* < 0.001), suggesting that cerebral infarction has a significantly worse prognosis. There was also a statistically significant difference in survival curves between patients with intracerebral hemorrhage and those with traumatic subarachnoid hemorrhage (*p* = 0.0029), indicating that the etiology of hemorrhagic stroke has important prognostic implications.

**Table 3:** Mortality rates by diagnosis category.

Diagnosis category	30-day mortality rate	90-day mortality rate	1-year mortality rate	3-year mortality rate
Cerebral infarction	15.9989%	20.0055%	26.6285%	30.7986%
Intracerebral hemorrhage	29.7260%	33.6986%	41.3699%	44.9315%
Non-traumatic subarachnoid hemorrhage	24.2105%	26.6667%	29.4737%	30.1754%
Traumatic subarachnoid hemorrhage	15.4930%	18.7793%	23.9437%	25.5869%

### 3.2 Predictive factors affecting the mortality risk of stroke patients

#### 3.2.1 Analysis of clinical indicators

**Figure 2:**
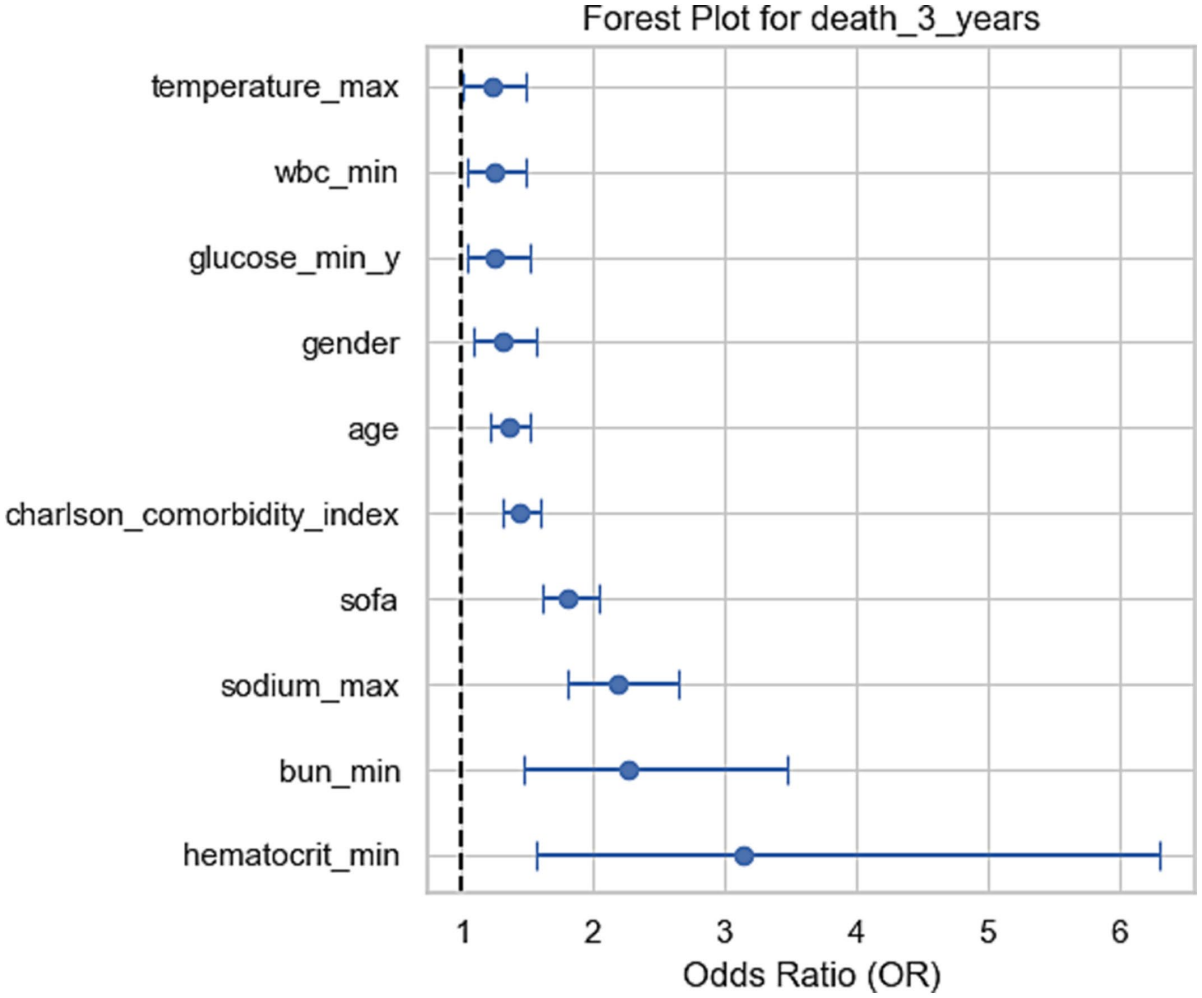
Forest plots for odds ratios (OR) of mortality at 3 years. These forest plots show the odds ratios (OR) of various clinical factors associated with mortality at 3 years. Each plot displays the OR and the 95% confidence intervals, indicating the relative impact of each factor on mortality at the corresponding time points. For the 3-year mortality, sodium, BUN, and hematocrit levels show significant associations with increased risk of death.

This study utilized a multiple logistic regression model to analyze a series of clinical indicators affecting the 30-day, 90-day, 1-year, and 3-year mortality risks in stroke patients (Table 4; Figure 2). The results revealed that weight was a statistically significant predictive factor; for each unit increase in weight, the mortality risk increased by 4,845.97 times (*p* = 0.0359) at 30 days, 1,862.99 times (*p* = 0.0457) at 90 days, and 1,124.31 times (*p* = 0.0441) at 1 year. This indicates that weight status may have clinical significance in both short-term and long-term outcomes. Furthermore, the lowest hemoglobin level was closely related to mortality risk. For every unit increase in the lowest hemoglobin level, the mortality risk increased by 3.33 times (*p* = 0.0053) at 30 days, 3.34 times (*p* = 0.0027) at 90 days, 4.16 times (*p* = 0.0002) at 1 year, and 3.14 times (*p* = 0.0013) at 3 years. This suggests that low hemoglobin can serve as an important biomarker for the prognosis of stroke.

**Table 4:** Comparison of survival curves between different stroke types using the log-rank test.

Comparison groups	*p*-value
Cerebral infarction vs. Intracerebral hemorrhage	0.0000
Cerebral infarction vs. Traumatic subarachnoid hemorrhage	0.4856
Cerebral infarction vs. Non-traumatic subarachnoid hemorrhage	0.7669
Intracerebral hemorrhage vs. Traumatic subarachnoid hemorrhage	0.0029
Intracerebral hemorrhage vs. Non-traumatic subarachnoid hemorrhage	0.1107
Traumatic subarachnoid hemorrhage vs. Non-traumatic subarachnoid hemorrhage	0.1797

The *p*-values were calculated using the Log-rank test to compare Kaplan–Meier survival curves between different stroke types.

Other indicators, including the highest sodium level, lowest blood urea nitrogen, and SOFA scores, also showed significant correlations with mortality risk. For every unit increase in SOFA score, the mortality risk increased by 2.11 times (*p* < 0.001) at 30 days, 2.03 times (*p* < 0.001) at 90 days, and 1.84 times (*p* < 0.001) at 1 year and 3 years respectively, highlighting the critical impact of organ dysfunction on prognosis. For each unit increase in comorbidity index, the mortality risk increased by 1.39 times (*p* < 0.001) at 1 year and 1.44 times (*p* < 0.001) at 3 years, indicating the significant role of underlying health conditions in long-term prognosis.

#### 3.2.2 Survival probability analysis

**Figure 3:**
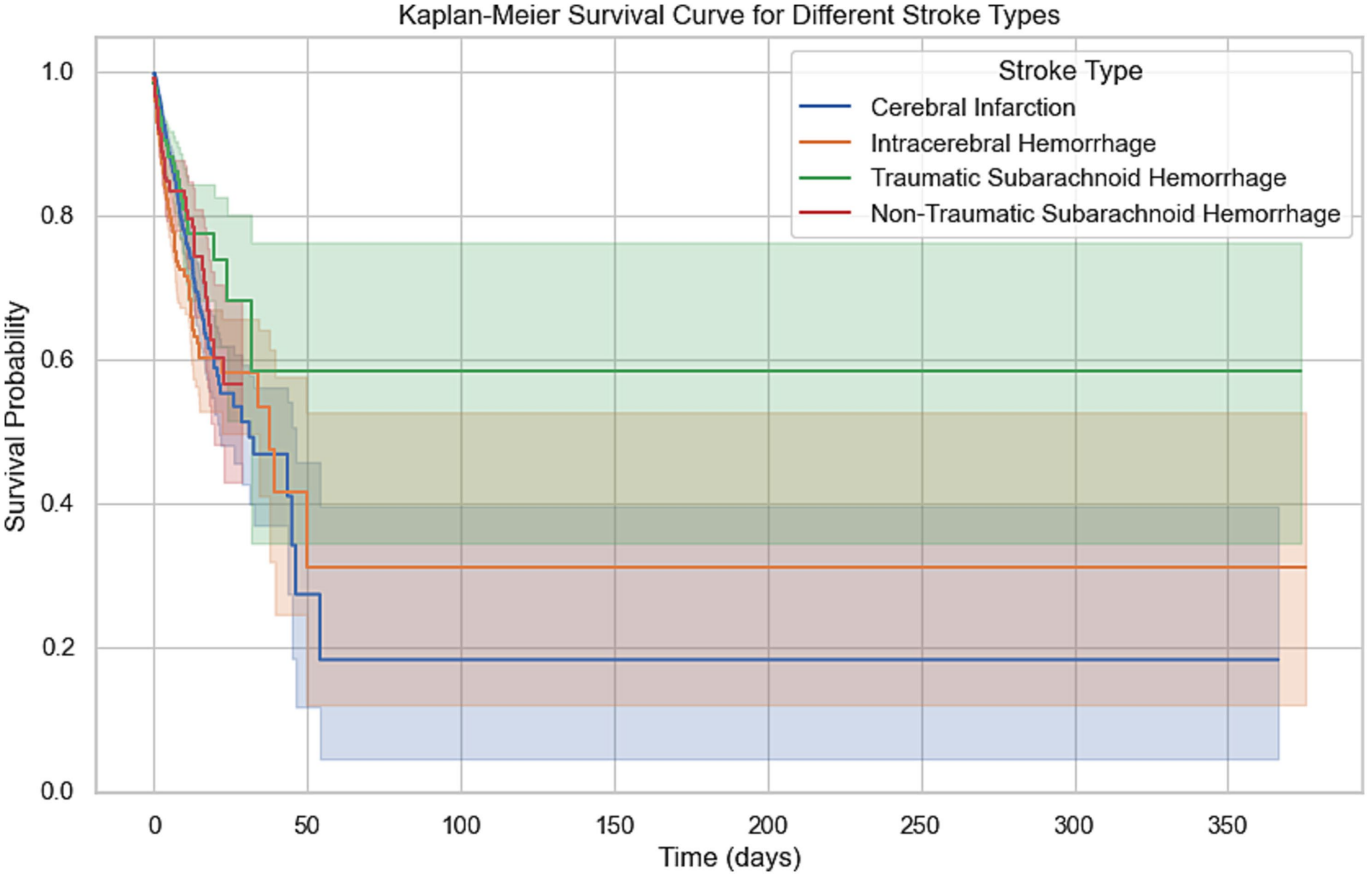
Kaplan–Meier survival curve for different stroke types. This Kaplan–Meier curve shows the 365-day survival probabilities for four stroke types: cerebral infarction, intracerebral hemorrhage, traumatic subarachnoid hemorrhage, and non-traumatic subarachnoid hemorrhage. The survival curves highlight the differences in survival trends, with shaded regions representing the 95% confidence intervals for each stroke type.

Kaplan–Meier survival analysis demonstrated significant differences in survival probabilities among different types of stroke patients during the 350-day follow-up period (Figure 3). The survival rate of patients with cerebral infarction was the lowest, with only about 20% surviving at 350 days; in contrast, patients with traumatic subarachnoid hemorrhage had the highest survival rate, with about 60% surviving at the end of follow-up. Survival rates for patients with intracerebral hemorrhage and non-traumatic subarachnoid hemorrhage were intermediate, at approximately 30 and 50%, respectively, at 350 days. The survival analysis demonstrated that age was a significant prognostic factor for patients with intracerebral hemorrhage and non-traumatic subarachnoid hemorrhage (*p* < 0.001). This finding highlights the importance of considering age when managing stroke patients, particularly for those aged 71 years and older, who may require more intensive monitoring and tailored treatment strategies. The severity of comorbidities was also a key prognostic indicator, with the survival rate of the Charlson comorbidity index ≥7 group being significantly lower than that of the 0–2 group (*p* < 0.001), 5–6 group (*p* = 0.0059), and 3–4 group (*p* < 0.001). The difference between the 5–6 group and the 3–4 group was also statistically significant (*p* = 0.0169), while there was no significant difference between the 0–2 group and the 3–4 group (*p* = 0.2087). To identify the most critical threshold for predicting survival decline, we performed a stratified analysis of the Charlson Comorbidity Index (CCI) at multiple thresholds (>2, >3, >4, >5, and > 6). For each threshold, we calculated the median survival time for the high-risk group (CCI above the threshold) and the low-risk group (CCI below or equal to the threshold), as well as the median survival time difference (Median Survival Difference). The results showed that CCI > 3 was the most critical cutoff point, with the largest median survival time difference (0.46). While CCI > 2 also demonstrated a substantial difference (0.45), thresholds beyond 4 showed minimal or even negative differences, indicating limited prognostic value. These findings suggest that CCI > 3 is the most significant threshold for predicting survival decline. The survival curves for different thresholds further supported this conclusion, as the separation between the high-risk and low-risk groups was most pronounced at CCI > 3 (Table 3; Supplementary Figure S1).

**Figure 4:**
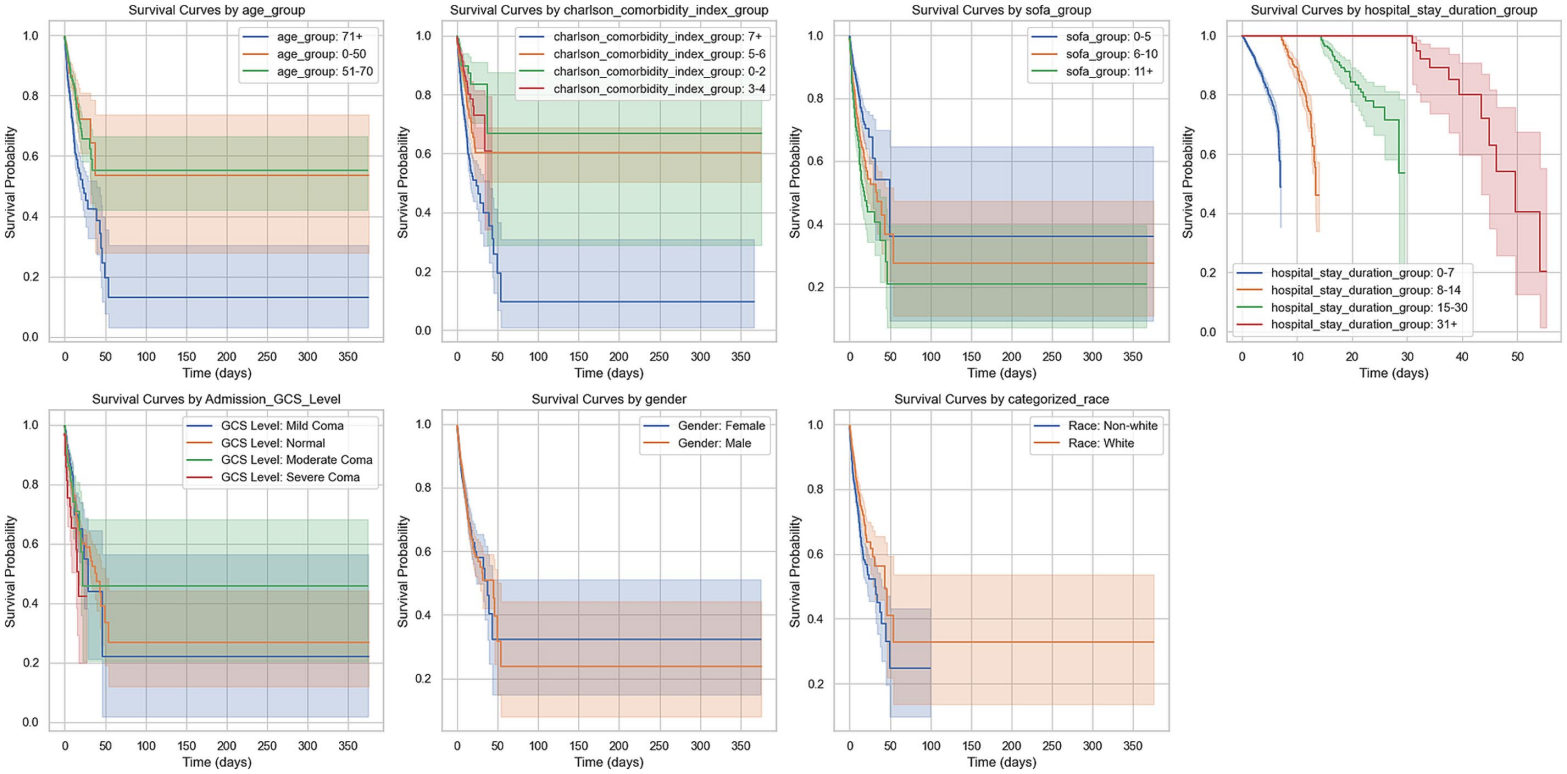
Kaplan–Meier survival curves by various clinical groupings. This figure presents Kaplan–Meier survival curves for different patient groups over 365 days. The groups include: Age groups (0–50, 51–70, 71+ years). Charlson comorbidity index groups (0–2, 3–4, 5–6, 7+). SOFA score groups (0–5, 6–10, 11+). Hospital stay duration groups (0–7, 8–14, 15–30, 31+ days). GCS level at admission (normal, mild coma, moderate coma, severe coma). Gender (male, female). Race (white, non-white). The curves show survival probabilities over time, with shaded areas indicating 95% confidence intervals. Survival trends differ across groups, with higher age, higher Charlson comorbidity index, and longer hospital stays generally associated with worse survival outcomes.

Higher SOFA scores correlated with worse prognosis; the survival curves between the 0–5 group and the 6–10 group (*p* < 0.001) and those above 11 points (*p* < 0.001) showed significant differences, with the difference between the 6–10 group and those above 11 points also being statistically significant (*p* = 0.0277). Additionally, the duration of hospitalization was another significant prognostic factor, with substantial differences between groups (*p* < 0.001). The neurological function status at admission also affected prognosis, with significant survival curve differences between the GCS 3 group and the GCS 1 group (*p* < 0.001) and the GCS 0 group (*p* = 0.0003), while no statistical difference was found between the GCS 1 group and other groups. Finally, race was also an important prognostic factor, with Caucasians showing significantly higher survival rates than non-Caucasians (*p* < 0.001) (Figure 4).

### 3.3 Predictive models and feature importance analysis for mortality risk in stroke patients

#### 3.3.1 Predictive model construction

**Figure 5:**
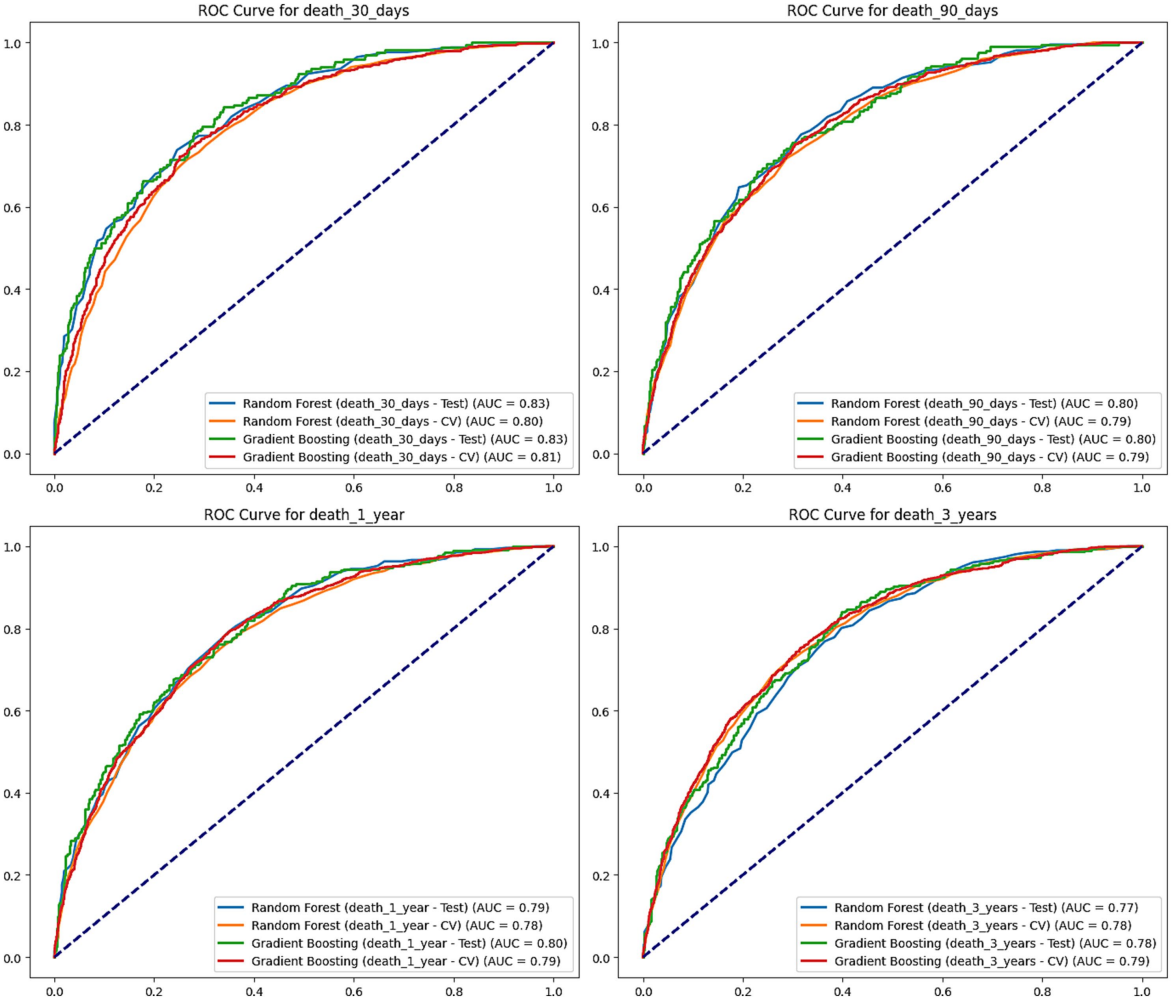
ROC curves for mortality prediction at 30 days, 90 days, 1 year, and 3 years. This figure shows the Receiver Operating Characteristic (ROC) curves for Random Forest and Gradient Boosting models predicting mortality at 30 days, 90 days, 1 year, and 3 years. The performance of each model is evaluated on both the test set and cross-validation (CV), with the area under the curve (AUC) values shown for each. Random Forest and Gradient Boosting models perform similarly across all time points, with AUC values ranging from 0.77 to 0.83, indicating good predictive performance.

This study utilized machine learning techniques to construct predictive models based on clinical characteristics of stroke patients to assess their mortality risks at 30 days, 90 days, 1 year, and 3 years (Figure 5). We employed two widely used algorithms, random forests and gradient boosting trees, and evaluated model performance through testing sets and cross-validation.

For 30-day mortality predictions, the random forest model had an AUC of 0.826 on the test set, while the gradient boosting tree model had an AUC of 0.833. In cross-validation, the AUC of the two models was 0.796 and 0.807, respectively. This indicates that both models can accurately predict the 30-day mortality risk of stroke patients in the short term.

For 90-day predictions, the random forest and gradient boosting tree models had AUCs of 0.802 and 0.804 on the test set, and AUCs of 0.787 and 0.794 in cross-validation, maintaining good predictive performance. In the predictions for 1-year and 3-year mortality risks, both models showed a slight decline in performance but still maintained high AUC levels. The AUCs of the random forest model on the test set were 0.794 and 0.768, while the AUCs for the gradient boosting tree model were 0.799 and 0.783. In cross-validation, the random forest AUCs were 0.778 and 0.783, with the gradient boosting tree AUCs being 0.787 and 0.788.

#### 3.3.2 Feature importance analysis

**Figure 6:**
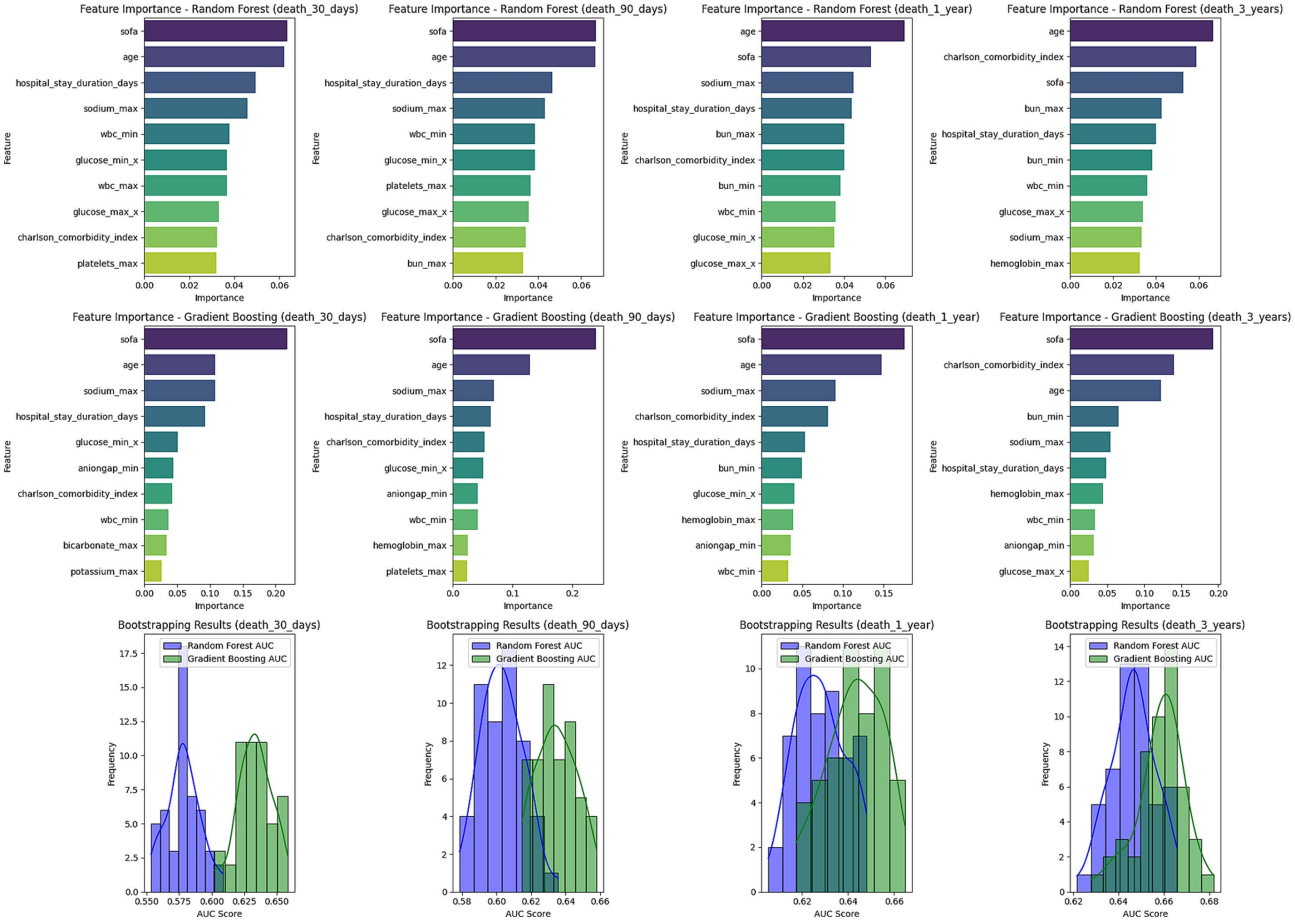
Feature importance and bootstrapping results for mortality prediction models. This figure presents the feature importance for Random Forest and Gradient Boosting models predicting mortality at 30 days, 90 days, 1 year, and 3 years. The top contributing factors include SOFA score, age, sodium levels, and hospital stay duration across all time points. Below the feature importance plots, bootstrapping results for AUC scores are shown for each model, illustrating the distribution of AUC values for Random Forest and Gradient Boosting. The bootstrapping results indicate model robustness across different time frames.

This study conducted a feature importance analysis using both the random forest and gradient boosting models to identify key factors influencing the 30-day, 90-day, 1-year, and 3-year mortality risks of stroke patients (Figure 6). The analysis results showed that age is a fundamental predictive factor, with importance scores for the random forest and gradient boosting models being 0.0621 and 0.1081, respectively, for 30-day mortality predictions.

Moreover, SOFA scores also played a critical role, particularly in the gradient boosting tree model, where the importance score for 30-day mortality prediction reached 0.2181, highlighting its significance in assessing prognosis. The Charlson comorbidity index showed high importance throughout the observation period, reflecting the cumulative impact of comorbidities on overall survival. The length of hospital stay also significantly impacted mortality risk predictions, particularly noted in the random forest model. Although the GCS score at admission contributed relatively less compared to other factors, it still provided relevant information for predicting mortality risk. These findings have significant implications for clinical decision-making and emphasize the importance of focusing on patient age, organ function status, and comorbidities in evaluating prognosis and formulating management strategies.

To validate the robustness of the models, we employed k-fold cross-validation and bootstrapping methods. The results showed that the predicted AUC values of both random forest and gradient boosting models ranged from 0.58 to 0.66 across different time periods, and the standard deviation of the bootstrapped AUC values was small, indicating good stability and reliability of the models. Notably, the gradient boosting model slightly outperformed the random forest model in predicting 1-year and 3-year mortality risks, suggesting its potential suitability for long-term risk modeling.

## 4 Discussion

This study utilized data from the MIMIC-IV database to assess key factors influencing short- and long-term mortality risks in stroke patients through a retrospective cohort analysis. The database includes hospitalized patient data from 2010 to 2020, and a total of 5,110 stroke patients aged 18 and older were included. The exposure variables comprised body weight, hemoglobin levels, electrolytes, kidney function, organ function scores, and comorbidity indices, while the outcome variables were the 30-day, 90-day, 1-year, and 3-year mortality risks. Long-term follow-up (a highlight of this study) revealed the association between these exposure variables and mortality risks in stroke patients.

The results demonstrated that the Charlson Comorbidity Index and SOFA score significantly impacted mortality risk, while lower hemoglobin levels were strongly associated with both short- and long-term mortality risks. Specifically, for each 1-point increase in the Charlson Comorbidity Index, the 1-year and 3-year mortality risks increased by 1.39 times (95% CI: 1.10–1.56) and 1.44 times, respectively. For each 1-point increase in the SOFA score, the 30-day, 90-day, 1-year, and 3-year mortality risks increased by 2.11 times, 2.03 times, and 1.84 times, respectively. Additionally, lower hemoglobin levels were significantly associated with increased mortality, with 1-year mortality risk increasing by 4.16 times (*p* < 0.005). These findings provide important insights for clinical risk assessment and emphasize the critical role of factors such as body weight, hemoglobin, and kidney function in stroke prognosis.

Our research findings are consistent with some previous studies while also presenting unique insights. Firstly, we confirmed that the Charlson Comorbidity Index, SOFA score, and hemoglobin levels are critical factors influencing the prognosis of stroke patients, which aligns with earlier findings. For instance, a prospective cohort study involving 1,013 acute ischemic stroke patients found a significant association between the Charlson Comorbidity Index and long-term prognosis, with each 1-point increase raising the 1-year mortality risk by 1.29 times (95% CI: 1.18–1.42) (12). This result is very close to our findings, although our study had a larger sample size and included different types of stroke patients.

In terms of the SOFA score, our research emphasized its significant role in predicting stroke patient outcomes. This conclusion is also supported by other studies. For example, a study focusing on severe acute ischemic stroke patients demonstrated that the SOFA score had high predictive value, with AUCs of 0.81 and 0.82 on days 4 and 7, respectively (13). Furthermore, another study developed a SOFA-based screening tool (S-SOFA) to identify non-ICU stroke patients at high risk of sepsis (14). These findings further underscore the importance of the SOFA score in stroke management.

Moreover, this study found that patients with ischemic stroke (cerebral infarction) had the poorest survival rates, a result that warrants further investigation. The poor survival in ischemic stroke may be attributed to several factors. First, from a pathological perspective, ischemic stroke is typically caused by vascular occlusion, leading to ischemia and hypoxia in brain tissue, resulting in irreversible brain damage and limited recovery potential of the affected tissue (15). In contrast, while hemorrhagic stroke (e.g., intracerebral hemorrhage) often causes more severe initial damage, some patients may exhibit recovery potential after the bleeding stops (16). Second, ischemic stroke patients are often burdened with more cardiovascular risk factors, such as hypertension, diabetes, and hyperlipidemia, which can exacerbate the condition and significantly impact prognosis (17). Finally, although advancements in treatments such as thrombolysis and mechanical thrombectomy have improved outcomes for ischemic stroke in recent years, the strict time window for these interventions and the relatively high failure rates may contribute to poorer long-term survival in some patients (18, 19). This finding aligns with previous research. For example, a cohort study of ischemic stroke patients reported that those with cardiovascular comorbidities had significantly reduced long-term survival rates (20).

However, our study also revealed some novel findings. We found that hemoglobin levels were closely associated with both short-term and long-term prognosis in stroke patients, a point that has not been fully addressed in previous research. For instance, a study comparing renal function in hemorrhagic and ischemic stroke patients primarily focused on the impact of renal dysfunction on prognosis without delving into the role of hemoglobin levels (21). This discrepancy may be due to differences in study design, as our research incorporated a more comprehensive set of variables and a longer follow-up period, enabling us to highlight the prognostic role of hemoglobin levels. This study highlights the significant impact of hemoglobin levels on the risk of mortality in stroke patients, a finding with important clinical implications. Low hemoglobin levels may adversely affect patient outcomes by exacerbating tissue hypoxia and increasing cardiovascular burden (22, 23). Therefore, it is recommended that hemoglobin level monitoring be incorporated into the routine assessment of stroke patients in clinical practice. Early interventions for patients with anemia, such as nutritional support, erythropoietin therapy, or blood transfusion when necessary, should be considered (24). Future research could further explore the dynamic changes in hemoglobin levels and the effects of targeted interventions to optimize personalized treatment strategies for stroke patients.

In addition to the Charlson Comorbidity Index and SOFA score, our study also highlighted the impact of age and length of hospital stay on prognosis. A large-scale retrospective study confirmed that age is a significant factor influencing long-term survival in stroke patients (25). Moreover, prolonged hospital stays were found to be associated with a higher risk of complications and poorer functional outcomes (12). These results provide further evidence that age and hospitalization factors should be considered when formulating treatment plans for stroke patients.

Mechanistically, our findings can be explained by the brain-kidney interaction theory. Stroke can lead to renal dysfunction, which in turn exacerbates brain injury, creating a vicious cycle (26). This theory supports the significant predictive value of the Charlson Comorbidity Index and SOFA score in our study. Hemoglobin levels, as an indicator of anemia and overall health, can also be explained through this mechanism, as anemia can worsen tissue hypoxia, thus influencing patient outcomes.

In the multivariable Cox regression analysis, we evaluated the interactions between key variables, including the SOFA score, Charlson comorbidity index, and other variables such as age and sex. Preliminary analysis indicated that these interaction terms did not have a significant impact on the outcome variable and were therefore excluded from the final model. We opted for the most parsimonious model to avoid overfitting while ensuring interpretability and robustness of the results (27, 28).

In this study, body weight demonstrated a statistically significant effect as a predictor; however, the relatively high odds ratio may be related to the choice of variable units. Since the odds ratio reflects the effect per unit change, using kilograms as the unit for body weight might have resulted in an inflated value. Additionally, body weight may indirectly influence prognosis as a surrogate marker for disease severity or nutritional status rather than exerting a direct independent effect. For instance, extremely low body weight may indicate malnutrition or chronic wasting diseases, while extremely high body weight may be associated with metabolic disorders, both of which could impact patient outcomes (29). Although the confidence interval was relatively wide, the statistical significance of body weight suggests that its effect on the outcome variable is robust. Future studies with larger sample sizes or stratified analyses are warranted to further validate the effect of body weight.

Another highlight of our study was the use of advanced machine learning methods such as random forests and gradient boosting tree models. These techniques provide more comprehensive and accurate predictions compared to traditional statistical methods. This approach has been applied in recent studies as well, such as a study that used machine learning to predict 90-day outcomes in acute ischemic stroke patients (30). This suggests that integrating emerging technologies in our research methodology offers promising support for more accurate prognostic assessments in future stroke studies.

Additionally, we evaluated the impact of electrolyte levels on stroke prognosis, which has been relatively overlooked in prior studies. For example, one study found a significant association between serum sodium levels and outcomes in acute ischemic stroke patients (31). This finding highlights the importance of considering a broader range of physiological parameters when assessing stroke patient prognosis.

Lastly, our study was based on the MIMIC-IV database, which provided a large sample size and multi-variable data, enabling a more comprehensive analysis. The advantages of this approach were demonstrated in a study that utilized electronic health records to predict stroke patient outcomes (32). By employing large-scale data analysis, we were able to more accurately identify key factors influencing prognosis, offering valuable insights for future clinical practice.

In summary, our research provides a more comprehensive perspective on stroke patient prognosis by considering multiple factors, including comorbidities, organ function, hematological indicators, and hospitalization circumstances. These findings not only validate prior research but also offer new insights, providing important evidence for risk assessment and personalized treatment strategy development in clinical practice. The clinical significance of this study lies in its provision of a comprehensive prognostic assessment framework for stroke, offering valuable insights for clinical practice. Through in-depth analysis of key factors such as the Charlson Comorbidity Index, SOFA score, and hemoglobin levels, we not only validated the importance of known risk factors but also uncovered novel predictive indicators. This multidimensional assessment approach surpasses traditional single-indicator predictions, providing a more precise basis for developing individualized treatment strategies. Our findings underscore the importance of early intervention, particularly for elderly patients with multiple organ dysfunction and severe anemia. Based on these results, we recommend conducting comprehensive risk assessments upon admission for stroke patients, including detailed comorbidity evaluations and organ function scoring, along with close monitoring of hemoglobin levels. Furthermore, the predictive model developed in this study can assist clinicians in more accurately assessing patients’ short-term and long-term mortality risks, thereby optimizing resource allocation and formulating more targeted treatment plans. Future research directions could explore the specific manifestations of these predictive factors in different stroke subtypes and develop AI-based real-time prediction tools to further enhance the accuracy and clinical applicability of predictions. In summary, this study provides important scientific evidence for improving the prognostic management of stroke patients and has the potential to drive updates and refinements in relevant clinical guidelines.

This study possesses several notable strengths. Firstly, we utilized a large-scale sample (*n* = 5,110) from the MIMIC-IV database, covering long-term data from 2010 to 2020, which enhances the reliability and representativeness of our findings. Secondly, we adopted a multidimensional approach to prognosis assessment, considering not only traditional clinical indicators but also comprehensive scores such as the Charlson Comorbidity Index and SOFA score, providing a more holistic risk assessment framework. In terms of data analysis strategy, we combined conventional statistical methods (e.g., Kaplan–Meier survival analysis and Cox regression) with advanced machine learning techniques (e.g., random forest and gradient boosting tree models). This innovative analytical approach not only validated known risk factors but also uncovered new predictive indicators. Particularly noteworthy is our simultaneous evaluation of both short-term (30-day, 90-day) and long-term (1-year, 3-year) prognoses, which is relatively rare in existing literature and provides a more comprehensive temporal reference for clinical decision-making. Furthermore, we conducted stratified analyses for different types of stroke, revealing detailed prognostic differences among subtypes, which is crucial for developing targeted treatment strategies. Lastly, our predictive model incorporates not only static factors but also dynamically changing clinical indicators (such as hemoglobin levels), enhancing the model’s predictive accuracy and clinical applicability. Overall, the design and analytical methods of this study provide a comprehensive, innovative, and practical paradigm for stroke prognosis research.

This study has several limitations. First, it only included patients aged 18 years and older, excluding pediatric and adolescent patients, which limits the generalizability of the findings to younger populations. Second, the data were derived from a single-center source (the MIMIC-IV database), which may restrict the generalizability of the results. Future multi-center studies are needed to validate our findings. Additionally, as an observational study, we can only detect associations between variables rather than establish causal relationships. Although we adjusted for measurable confounding factors, the potential influence of unmeasured confounders cannot be completely ruled out. It is also worth noting that the MIMIC-IV database primarily consists of data from U.S. patients, which may limit the applicability of the findings to other racial and geographic populations. Caution should be exercised when applying our results to other populations, considering potential racial and regional differences. Lastly, due to the limitations of the database, we were unable to access certain important clinical information, such as patients’ lifestyle factors, family history, and specific treatment details, which could significantly impact prognosis. While the random forest and gradient boosting tree models demonstrated excellent predictive performance, their application in real-world clinical practice remains challenging, particularly due to the complexity of the models and their reliance on data quality. Future research could explore the development of real-time prediction tools based on artificial intelligence to enhance the clinical applicability of such models. Despite these limitations, this study provides valuable insights into understanding stroke prognostic factors and paves the way for future research.

In conclusion, this comprehensive study utilizing the MIMIC-IV database has identified key prognostic factors for stroke patients, including the Charlson Comorbidity Index, SOFA score, and hemoglobin levels, which significantly impact both short-term and long-term mortality risks. These findings provide valuable insights for clinical risk assessment and emphasize the importance of multidimensional evaluation in stroke management, potentially leading to more personalized and effective treatment strategies.

## Data Availability

The raw data supporting the conclusions of this article will be made available by the authors, without undue reservation.

## Supplementary material

SUPPLEMENTARY FIGURE S1 Survival Curves by Charlson Comorbidity Index (CCI) Thresholds. Kaplan-Meier survival curves show the impact of different CCI thresholds on survival probabilities. The analysis identifies CCI > 3 as the most critical threshold, with the largest median survival time difference (0.46), indicating its strong discriminatory ability for survival outcomes. Thresholds > 4 and higher show a diminished impact on survival.
