# Supplementary figures and images for: Retrospective cohort study based on the MIMIC-IV database: analysis of factors influencing all-cause mortality at 30 days, 90 days, 1 year, and 3 years in patients with different types of stroke
Source: Front Neurol. 2025 Jan 7;15:1516079. doi: 10.3389/fneur.2024.1516079 (PMC11746016; doi:10.3389/fneur.2024.1516079)

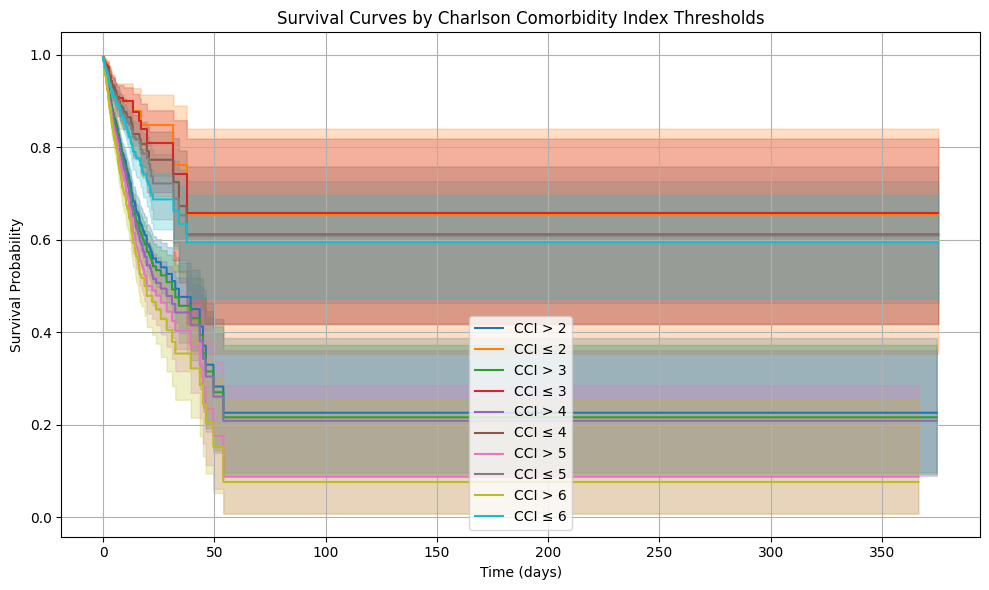

Supplement: SUPPLEMENTARY FIGURE S1 — Survival Curves by Charlson Comorbidity Index (CCI) Thresholds. Kaplan-Meier survival curves show the impact of different CCI thresholds on survival probabilities. The analysis identifies CCI > 3 as the most critical threshold, with the largest median survival time difference (0.46), indicating its strong discriminatory ability for survival outcomes. Thresholds > 4 and higher show a diminished impact on survival. [file Image_1.tif]
